# Apoptotic mimicry as a strategy for the establishment of parasitic infections: parasite- and host-derived phosphatidylserine as key molecule

**DOI:** 10.1186/s12964-019-0482-8

**Published:** 2020-01-15

**Authors:** João Luiz Mendes Wanderley, Renato Augusto DaMatta, Marcello André Barcinski

**Affiliations:** 1 Laboratório de Imunoparasitologia, Campus UFRJ Macaé, Universidade Federal do Rio de Janeiro, Rio de Janeiro, RJ Brazil; 2 Laboratório de Biologia Celular e Tecidual, Centro de Biociências e Biotecnologia, Universidade Estadual Norte-Fluminense, Campos dos Goytacazes, RJ Brazil; 3 Instituto de Biofísica Carlos Chagas Filho, Universidade Federal do Rio de Janeiro, Rio de Janeiro, RJ Brazil

**Keywords:** Phosphatidylserine, Apoptotic mimicry, Parasites, Host-parasite interplay

## Abstract

The establishment of parasitic infection is dependent on the development of efficient strategies to evade the host defense mechanisms. Phosphatidylserine (PS) molecules are pivotal for apoptotic cell recognition and clearance by professional phagocytes. Moreover, PS receptors are able to trigger anti-inflammatory and immunosuppressive responses by phagocytes, either by coupled enzymes or through the induction of regulatory cytokine secretion. These PS-dependent events are exploited by parasites in a mechanism called apoptotic mimicry. Generally, apoptotic mimicry refers to the effects of PS recognition for the initiation and maintenance of pathogenic infections. However, in this context, PS molecules can be recognized on the surface of the infectious agent or in the surface of apoptotic host debris, leading to the respective denomination of classical and non-classical apoptotic mimicry. In this review, we discuss the role of PS in the pathogenesis of several human infections caused by protozoan parasites.

Video Abstract

Video Abstract

## Background

### Apoptosis and apoptotic mimicry

Parasites have to cope with the host immune system to establish infection. These organisms acquire evolutive adaptations to evade, inhibit or deviate the immune response, aiming to turn the host permissive to infection maintenance and dissemination. In several systems, parasites make use of host molecules to achieve this goal or display their own immune-modulating molecules. The observation of apoptotic death or apoptotic–like phenotypes in parasites raised several questions regarding the consequences of such mechanism operating in unicellular parasitic organisms [1–4]. Currently, it is well established that apoptotic cells or apoptotic–derived molecules play a role in the establishment and the outcome of different parasitic infections. This mechanism was first demonstrated in 2001, when it was observed that amastigote forms of *Leishmania amazonensis* are able to expose phosphatidylserine (PS) in the outer leaflet of the plasma membrane, and that this molecule is important for amastigote infection and maintenance of the consequent experimental leishmanial disease [5]. PS is one of the main early-stage apoptotic molecules displayed by dying cells [6]. PS exposure occurs due to a caspase-dependent plasma membrane asymmetry loss, caused by the cleavage of phospholipid translocases [7, 8]. Once in the cell surface, PS recognition by epithelial and immune cells triggers the endocytosis of the target cell, as well as the activation of anti-inflammatory and immunosuppressive responses by the phagocyte [9, 10].

The effects of PS recognition in the regulation of local and systemic inflammation and the promotion of immune tolerance are advantageous for parasite establishment and dissemination, independently on the source of PS. Following the demonstration of apoptotic mimicry in an experimental model of infection by *L. amazonensis*, several papers reported that different versions of the apoptotic mimicry operate in assorted models, such as in other parasite and viral infections and in tumor development [11–13]. In all these cases, PS recognition is involved in the pathogenesis and maintenance of the disease caused by those infective agents and tumor cells. The better understanding of the role of PS in these events led to the description of two distinct patterns of apoptotic mimicry: classical and non-classical apoptotic mimicry [12]. Classical apoptotic mimicry ensues when the source of PS is the plasma membrane of the organism or cell taking advantage of PS recognition. This mechanism operates in (a): enveloped viral infections. Several viruses are able to invade and deactivate host cells through PS on their envelope surface [11, 12, 14–16], (b): tumor development. Tumor cells and shed microvesicles display PS on their surface, promoting tumor spreading, immune tolerance and endothelial cell activation [11, 13, 17–19], and (c) parasite infections, which are the focus of the present review. On the other hand, non-classical apoptotic mimicry occurs when pathogens or tumor cells take advantage of PS exposed by host cells, either by inducing cell death in host cells, or following natural death due to host cell activation or inflammation. This is the case of some non-enveloped viruses [12] and parasites.

In this review we propose to discuss the role of PS in the context of both classical and non-classical apoptotic mimicry, on the perspective of different parasite infections of interest for human health.

### Classical apoptotic mimicry

#### Definition

Apoptotic mimicry was first demonstrated as a strategy employed by intracellular parasites in which exposed PS acts as a signal for parasite internalization in host cells and induces an anti-inflammatory response. PS translocation to the outer leaflet of the plasma membrane can occur transiently in several circumstances, such as T cell, mast cell and platelet activation, myotube formation and endothelial cell inflammatory stimulation [20–25]. However, constitutive and irreversible PS translocation is a characteristic of early apoptotic cells and is usually followed by cell death [6, 7, 26, 27]. In the original description of apoptotic mimicry, viable *L. amazonensis* amastigotes were shown to expose PS as a strategy to persist in the host [5]. The main impact of this mechanism was the decrease in nitric oxide (NO) production by infected macrophages. NO is the main macrophagic microbicidal molecule with activity against parasites, since it is capable of inactivate several metabolic enzymes by nitrosylation reactions [28]. Currently, in addition to the original description, it is well established that PS exposed on viral particles, tumor cells and, particularly, protozoan parasites can play a similar role [11]. Thus, in the first part of this review we will discuss the role of PS molecules in classical apoptotic mimicry performed by parasites of importance in human diseases.

#### Leishmania amazonensis

Parasites of the genus *Leishmania* are the causative agents of leishmaniasis, a neglected disease that affects 1,3 million people, mostly in tropical and subtropical countries, leading to 20.000 deaths per year. It is estimated that over 1 billion people live in endemic areas at risk of infection [29]. These protozoan organisms are heteroxenic parasites that infect phlebotomine sandfly vectors and mammalian hosts, including humans. Promastigote forms survive in the intestinal tract of phlebotomines and differentiate into metacyclic promastigotes, the infective stage for mammalian hosts. When deposited in the lesion during blood feeding, metacyclics are able to resist the innate immune system and infect phagocytic cells, differentiating into non-motile, rounded amastigotes. These forms are able to proliferate inside parasitophorous vacuoles in the host cell, adding to cell disruption, infection of new host cells and dissemination [30, 31].

**Fig. 1:**
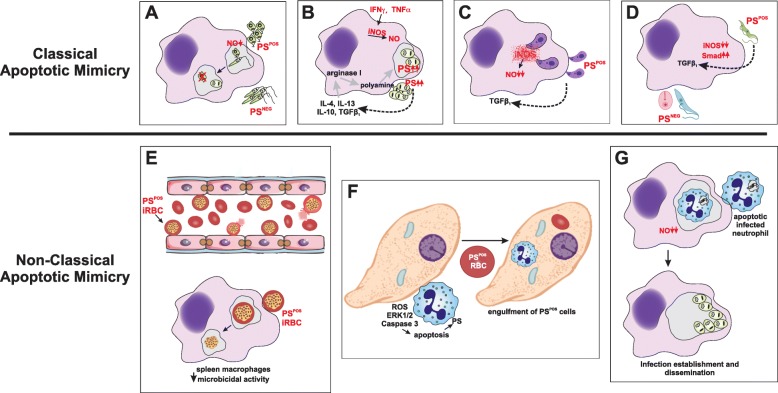
Classical and Non-classical apoptotic mimicry. Apoptotic mimicry employed by intracellular parasites to establish and maintain infection. **a** PS^POS^ *Leishmania* spp. promastigotes from in vitro cultures or from phlebotomine sandflies are necessary to establish infection, due to their ability to inhibit NO production on infected macrophages. **b** Intracellular *L. amazonensis* amastigotes are able to expose PS at their surface. PS exposure is induced and maintained by the concomitant activity of arginase and iNOS. PS exposure on these parasites is a counteraction for macrophage microbicidal activity. **c** *Toxoplasma gondii* tachyzoites are able to expose PS and macrophage infection with these parasites led to iNOS degradation and parasite establishment. **d** *Trypanosoma cruzi* trypomastigotes are the infective forms produced by infected mosquitoes. These forms are the only ones capable of expose PS and establish infection due to a TGFβ-dependent inhibition of iNOS expression. Epimastigotes and amastigotes do not expose PS. **e** Red blood cells infected with *Plasmodium* spp. (iRBC) are able to adhere to endothelial cells, promoting parasite resistance in the peripheral blood. In addition, spleen macrophages are able to engulf iRBC, leading to parasite persistence inside these splenic cells. **f** *Entamoeba histolytica* trophozoites induce the apoptotic death of neutrophils (depicted here), hepatocytes, endothelial and epithelial cells. PS exposure on apoptotic cells lead to the engulfment of these cells by trophozoites, which has an impact in trophozoite nutrition and the ability to invade host tissues. In addition, trophozoites are able to engulf naturally PS^POS^ red blood cells, leading to similar effects. **g** Infected neutrophils are able to undergo apoptosis, either by physiological or infection induction. In both cases, apoptotic infected neutrophils are engulfed by macrophages, leading to macrophage alternative activation and parasite persistence and dissemination

The observation of PS exposure in *L. amazonensis* parasites was made when promastigote forms were submitted to a heat shock by transferring them from 23 °C to 37 °C, mimicking the temperature shift during a natural infection. Most promastigotes under these stressful conditions lose their viability, normal morphology and energetic metabolism [32]. Amastigote forms are adapted to the higher temperatures and lower pH encountered in mammalian hosts; therefore, they should not display apoptotic features in these conditions [33, 34]. However, when purified from mice lesions or macrophages in in vitro cultures, amastigotes expose PS in the outer leaflet of the plasma membrane, despite the maintenance of viability, morphology, and ability to infect other cells, animals, phlebotomine sandflies and to differentiate into promastigote forms [5, 35–37]. This observation led to the question whether PS exposure on amastigotes plays a role in the normal biology of the parasite, unrelated to cell death. It was observed that the recognition of PS on the surface of these parasites is fundamental for amastigote uptake by macrophages and, most importantly, to induce a permissive status in the host cell, allowing parasite intracellular growth and maintenance [5, 35–37]. During amastigote infection, PS is able to induce TGF-β1 and IL-10 production by macrophages, which decreases NO production [5, 35, 36]. Actually, the events triggered by PS recognition on amastigotes are similar to the ones observed during apoptotic cell recognition, as depicted in Fig. 1a. This similarity inspired the apoptotic mimicry concept to describe the phenomenon [5].

It is well established that susceptibility and resistance to *Leishmania* infection depends on the genetic background of the host, which, in turn, defines the properties of the anti-parasite immune response [31]. The first observation of apoptotic mimicry in amastigotes was made in the BALB/c mouse model of infection. BALB/c mice are susceptible to the infection with most of *Leishmania* species [31, 38]. In *L. amazonensis* infection, when amastigotes are obtained from mice strains with different susceptibility profiles, it was observed that PS exposure also varies, and directly correlates with host susceptibility [35], indicating that PS exposure on the amastigote is regulated by host intrinsic factors. Amastigotes purified from different mice strains and, therefore, with different amounts of PS exposed, maintain their distinctive infection capacity when infecting an unrelated host confirming that the amount of PS exposed defines the infective capacity of these parasites [35]. Interestingly, it was observed that an intrinsic pressure from the immune system is responsible to induce PS exposure on intracellular amastigotes. Amastigotes obtained from infected immune-deficient mice do not expose PS at their surface and the adoptive transfer of immune-competent primed T lymphocytes revert this phenotype [36]. Experimental evidences suggest that NO synthesis pathway is responsible for the induction of PS exposure on intracellular amastigotes, since the immune activation of infected iNOS (inducible nitric oxide synthase) KO macrophages does not stimulate PS exposure on the intracellular parasites. In addition, PS-dependent induction of polyamine synthesis by infected macrophages protects PS-exposing amastigotes from death. Infected macrophages that induce PS exposure on the intracellular parasites must maintain detectable levels of iNOS and arginase 1 expression [36]. Therefore, PS exposure is an adaptive response of intracellular amastigotes of *L. amazonensis* that counteracts the immune activation of the host cell. The biochemical mechanisms that regulate the translocation of PS in the parasite need to be determined, although there are some reports indicating that PS exposure in *Leishmania* parasites may be independent on metacaspase activation, dependent on endoplasmic reticulum oxidative stress in some species or dependent on caspase-like activity [39–42]. In addition, it is necessary to elucidate whether PS exposure on amastigotes occurs in different *Leishmania* species and its role for the establishment of the infection.

As already stated, PS-exposing promastigotes are undergoing apoptotic death and, therefore, are not viable [43–45]. However, PS-exposing promastigotes do cooperate with viable parasites to establish infection (Fig. 1b). The population of infective promastigotes derived from in vitro cultures and from phlebotomine sandflies contains a significant percentage of apoptotic, non-viable, PS-exposing parasites [44, 45]. The removal of the non-viable, PS-exposing parasites from the infective inoculum, decreases and, depending on the efficiency of the purification method, can even abrogate its infective capacity [44, 45]. The lack of nutrients in the culture media or in the intestinal tract of the sandfly is one of the signals that drive the differentiation of metacyclic promastigotes [46]. It is possible that a sub-population of promastigotas, unable to cope with these conditions, dies by apoptosis exposing PS in the process. Apoptotic metacyclic promastigotes are able to modulate macrophage activation allowing the establishment of the viable parasites. This mechanism was shown to operate in different *Leishmania* species, such as *L. major*, the main species related to leishmaniasis in the Mediterranean Europe, western Asia and north Africa and *L. amazonensis*, the causative agent of diffuse and disseminated leishmaniasis in the America [44, 45].

#### Trypanosoma cruzi

Chagas disease affects about 8 million people in the world [47] and is caused by the protozoan *Trypanosoma cruzi* that exists in three basic forms: amastigotes, trypomastigotes and epimastigotes. Trypomastigotes are released in the invertebrate vector feces and gain access to the mammalian host at the site of the vector bite or through a mucosal entry, infect host cells and differentiate into amastigotes that multiply and differentiate back into trypomastigotes. These forms are eventually drawn by the blood sucking vectors and in their gut differentiate into epimastigotes that turn back into trypomastigotes in the rectum of the vector [47].

All three forms have been studied to better understand the biology of the parasite. Epimastigotes are replicative forms that are killed by vertebrate host cells. Amastigotes are replicative intracellular forms, found in the vertebrate host cells that are capable of new infections and trypomastigotes are infective forms unable to replicate and when released by the vector, needs to cope with the vertebrate immune system [48]. PS exposure has been analyzed in all three forms of the parasite [49]. About 50% of trypomastigotes from mice blood or obtained from Vero cultures are able to expose PS at the outer leaflet of their plasma membrane. Epimastigotes and amastigotes do not to expose PS. When trypomastigotes differentiate into epimastigotes, PS exposure is abolished. In addition, trypomastigotes are able to trigger a TGF-β1 signaling pathway, leading to a decreased expression of iNOS in infected activated mice macrophages [49], as described in Fig. 1c. Thus, only the form that interacts with host macrophages, during the establishment of the infection, exposes PS.

#### Toxoplasma gondii

*Toxoplasma gondii* is an obligatory intracellular parasite of the phylum apicomplexan and is the etiological agent of toxoplasmosis. In humans, toxoplasmosis is one of the most recurrent infections infecting around 1/3 of the world’s population [50]. *T. gondii* was the second described protozoan capable of employ classical apoptotic mimicry [51]. Exposure of PS to the outer surface of *T. gondii* plasma membrane induces macrophage to release TGF-β1 leading to a decreased iNOS expression and reduced NO production [51]. The lower NO production decreases host cell microbicidal function and thus enhances parasite survival. We have recently shown that the mechanism of inhibition of NO production differs in distinct macrophage cell lines: iNOS is degraded in peritoneal macrophage-like lineage but remains expressed in RAW 264.7 lineage [52]. Santos et al. [53] isolated two subpopulations of *T. gondii*: the PS^POS^ subpopulation exposes PS in the outer plasma membrane layer and performs apoptotic mimicry, whereas the PS^NEG^ subpopulation display no such feature. Analysis of vacuoles formed after host cell invasion by these subpopulations reveals that PS^POS^ *T. gondii* are located in narrow vacuoles, indicating active penetration (Fig. 1d). In contrast, PS^NEG^ subpopulation of *T. gondii* dwells in large vacuoles, indicative of phagocytosis [54]. The PS^POS^ subpopulation of *T. gondii* is the only one capable of actively penetrating non-phagocytic host cells and inhibiting NO production following activated macrophage infection (Fig. 1d). However, in in vivo infection with a mixture of both PS subpopulations promotes longer survival in mice than infection with isolated subpopulations. Infection with PS^POS^ *T. gondii* triggers high parasite burden identified in animal tissue samples. In contrast, PS^NEG^ *T. gondii* induce an exacerbated inflammatory process. In both cases, the viability of mice is compromised [53].

### Non-classical apoptotic mimicry

#### Definition

Death of host cells, either by direct effects of the infective agent or as a consequence of inflammatory cell activation are possible features of a cycle of infection [55–58]. The accumulation of apoptotic cells and apoptotic bodies defines the outcome of the inflammatory and immune responses, contributing to the development of a tolerogenic environment [59–62]. In this case, parasites, viruses and possible other pathogens can coopt PS exposed by host dying cells as a strategy to create an environment that allows the infection to establish and to disseminate with effects on the disease severity and maintenance [55–58]. Therefore, in the last part of this review, we will discuss the consequences of PS exposing by host-derived cells for parasitic infections, the so called non-classical apoptotic mimicry [12].

#### Plasmodium spp.

*Plasmodium* is a genus of the apicomplexan phylum with more than 100 species that infect reptiles, birds and mammals [63] and has species of the *Anopheles* mosquitoes as vectors [64]. Humans are infected by 5 species of *Plasmodium* parasites (*P. falciparum*, *P. vivax*, *P. malariae*, *P. ovale* and *P. knowlesi*) that cause a disease known as malaria. This is a world-spread disease, with severe complications and significant morbidity and mortality and with no available vaccine. The cycle of these parasites is complex, with an exoerythrocytic and an erythrocytic phase. The erythrocytic phase involves the infection of erythrocytes (red blood cells - RBC) by the merozoite stage, that differentiate into ring stage, followed by the trophozoite stage, shizogony and the release of new merozoites with the rupture of RBC [65]. The erythrocytic phase of the cycle consumes RBC causing anemia, which is one of the clinical manifestations of malaria. Infection of RBC by *Plasmodium* spp. causes a considerable stress in these host cells interfering with their normal life span.

Normal RBCs age in the circulation and end up being removed and degraded from the circulation and degraded by spleen macrophages [66]. Aged RBCs exposes PS as one of the main “eat me” signal [67]. RBCs can also suffer life-threatening damage during its life course, leading to programmed cell death known as eryptosis. This type of cell death may be caused by different cellular stress that also induces the exposure of PS and consequent phagocytosis by spleen macrophages [68]. In addition, it has been reported that *Plasmodium* spp. infected RBCs (iRBCs) suffer great stress and expose PS. Treatment of mice with compounds that induce eryptosis, in different models of rodent experimental malaria, results in higher PS exposure of iRBCs and lower parasitemia This indicates that induced eryptosis may be an interesting strategy to treat malaria, although, the direct effect of the compound on intracellular RBC parasites cannot be ruled out [69] Nevertheless, it is not clear how PS exposure by iRBC affects the parasite or the host [69]. Interestingly, exposure of PS by *Plasmodium*-iRBCs may help parasite clearance from blood by spleen macrophages [70]. Alternatively, PS exposed by iRBC may turn these cells into “Trojan horses”, since PS exposure by iRBC induces uptake by macrophages, and at the same time may disarm the microbicidal capacity of these host cells due to the induction of anti-inflammatory responses [71]. Macrophage deactivation may lead to parasite persistence (Fig. 1e), which corroborates with latent merozoites forms found in the spleen of infected mice [72] and in mice dendritic cells [73].

PS is exposed by *P. falciparum*-iRBC in in vitro cultures [74, 75] and may be related to cytoadherence to vascular endothelium [76]. The role of PfEMP1 (*P. falciparum* erythrocyte membrane protein 1) on cytoadherence by *P. falciparum*-iRBC has been demonstrated [77], but cytoadherence of iRBC involves distinct cell surface receptors [77], suggesting that PS exposure by iRBCs may also be involved. Cytoadherence, which avoids parasite removal from circulation is part of the physiopathology of malarial infection and eventually contributes to host death (Fig. 1e). PS exposure of iRBCs has been demonstrated in humans with *P. falciparum* infection [78] and in mice infected with *P. yoelii* [79] and *P. berghei* [80]. Although PS exposure has not been demonstrated in *P. vivax*-iRBC it is possible that this mechanism plays a role in human infections by this parasite [76].. Cytoadherence, which avoids parasite removal from circulation is part of the physiopathology of malarial infection and eventually contributes to host death. Furthermore, it has been demonstrated that febrile temperatures induce higher PS exposure in *P. falciparum*-iRBCs suggesting a correlation between severity and PS exposure on iRBCs in this disease [81]. On the other hand, PS exposure by iRBC may increase their phagocytosis by spleen macrophages (Fig. 1e), removing parasites from the circulation, but also causing anemia [82, 83]. In addition, it has also been demonstrated that patients with uncomplicated *P. falciparum* and *P. vivax* malaria of display high levels of anti- phospholipid antibodies including anti-PS which may favor iRBC opsonization and parasite clearance [84]. Furthermore, it has been recently demonstrated that malaria patients infected with *P. vivax*, *P. falciparum*, *P. knowlesi* and *P. malariae* have higher IgM and IgG anti-PS antibody levels, when compared with healthy controls. The levels of anti-PS antibodies correlate with severity of patient’s anemia [85]. This is especially true in patients bearing *P. vivax* infections. These facts reinforce the evidences that PS exposed in RBC of infected patients have important, implications in the pathogenesis of malarial infections. The clearance by macrophages of antibody opsonized PS exposed on iRBC exposing PS would involve FC receptors, avoiding the anti-inflammatory response caused by PS [71], which would be advantageous to the host. Finally, the treatment of mice, in different models of rodent experimental malaria, with compounds that induce eryptosis, results in higher PS exposure of iRBCs and lower parasitemia, indicating that induced eryptosis may be an interesting strategy to treat malaria, although, the direct effect of the compound on intracellular RBC parasites cannot be ruled out [69]. In summary, *Plasmodium* spp. infection of RBC enhances PS exposure, but the physiological role of this exposure remains to be elucidated by further in vivo studies.

#### *Entamoeba histolytica*

Amebiasis is a human disease caused by protozoan parasites of the genus *Entamoeba*. Some species can be found as commensal organisms in the intestinal tract. However, when they develop an invasive phenotype they can penetrate the tissues causing dysentery, colitis and liver abscesses [86, 87]. Invasion of host tissues and disease promotion is correlated with the ability of the parasite to kill and engulf host cells. Actually, *Entamoeba histolytica*, the main pathogenic species, is able to induce cell death in neutrophils, epithelial cells, lymphocytes and hepatocytes, both in in vitro and in vivo conditions [88–90]. Trophozoites of *E. histolytica* are able to trigger a NADPH-dependent production of reactive oxygen species (ROS) in host cells, which induces the ERK1/2 and caspase 3-dependent apoptotic death of the host cell [90]. The exact mechanism by which these parasites are able to induce the apoptotic death of host cells is still unknown. Cell killing is dependent on contact mediated by lectins expressed by the parasite, interacting with N-acetylgalactosamine containing proteoglycans expressed by host cells [88]. Liver abscesses and hepatic failure caused by *E. histolytica* infections can be prevented by treating infected mice with the pan-caspase inhibitor zVAD-fmk, indicating that apoptosis of host cells plays a role in pathogenesis and disease severity [91, 92]. In addition to inducing apoptotic death of host cells, virulence of *E. histolytica* is dependent on the ability of these parasites to engulf host cells. This process is used to identify pathogenic ameba in the gut, since it is possible to observe engulfed host cells inside the parasites in cytological analysis of gut content [88, 90, 93]. PATMK, a transmembrane kinase protein has been identified as a receptor at the surface of trophozoites that participates in the engulfment of apoptotic cells [91]. The main ligand recognized by trophozoites of *E. histolytica* is PS. The ability of these parasites to recognize and engulf erythrocyte and lymphocytes is correlated with PS exposure by the host cells [89, 94]. Annexin V is able to inhibit ameba erythrophagocytosis up to 70% [93]. Transfer of PS to viable lymphocytes turns these cells targets for trophozoite phagocytosis, in a specific manner since transfer of phosphatidylethanolamine or phosphatic acid are unable to induce lymphocyte engulfment [88]. The stimulation of phagocytic of *E. histolytica* by PS has been already demonstrated in studies with liposomes derived from erythrocyte membranes. It was observed that liposomes possessing negatively charged phospholipids induce actin polymerization and trophozoite engulfment of targets [88]. In addition, *E. histolytica* express a calcium-dependent receptor named EhCaBP3 (*E. histolytica* calcium binding protein 3) that binds directly to PS and modulates cytoskeleton activity, mediating the phagocytosis of cellular corpses [95]. The exact consequence of the PS-dependent phagocytosis of host cells by *E. histolytica* trophozoites need to be further determined. However it has been clearly shown that highly pathogenic strains capable of inducing severe amebiasis are the ones adapted to induce apoptotic host cell death, recognize the “eat-me” PS-dependent signal and engulf the dead corpses [96, 97]. It is possible to hypothesize that these events may contribute to parasite nutrition, regulation of inflammation and disruption of cell barriers that prevent parasite invasion.

#### Trypanosoma cruzi

In addition to the already discussed role for PS exposed by trypomastigotes of *T. cruzi*, these parasites also make use of PS-derived signals from host cells. During *T. cruzi* experimental infection, lymphocytes show a dramatic increase in apoptotic cell death, upon activation with mitogens such as concanavalin A or anti-TCR αβ agonist antibodies. This mechanism seems to be due to T cell exhaustion caused by chronic T cell stimulation triggered by chronic infection [98]. The interaction between apoptotic lymphocytes and *T. cruzi* infected macrophages increase the growth of the parasite in a TGF-β1, prostaglandin and polyamine dependent way. In addition, transfer of apoptotic lymphocytes to infected mice increases parasitemia and this effect can be abolished by treatment with cyclooxygenase inhibitors, suggesting an important role of prostaglandins to increase infection [99]. These seminal results indicate that infection could increase apoptosis of critical immune cells and deactivate the immune system on behalf of the parasite, clearly demonstrating how a non-classical apoptotic mimicry operates.

#### Leishmania spp.

Besides the fact that both, promastigotes [44, 45] and amastigotes [5, 35], of *Leishmania* are able to employ classical apoptotic mimicry to establish infection, these parasites can also hijack host sources of PS, leading to persistence and dissemination, in a modified version of non-classical apoptotic mimicry. In natural and experimental infection, the earlier cells arriving at the infection site are neutrophils [100]. These cells are attracted by both inflammatory and phlebotomine salivary signals [101]. These cells can harbor *Leishmania* parasites, but they are not efficient as *Leishmania*- host cells. Intracellular differentiation and proliferation of amastigotes generally is not efficient, and tissue-infiltrating neutrophils do not survive long enough to maintain infection [102, 103]. However, the ability of promastigotes to infect and survive inside neutrophils permits these parasites to escape host innate protective mechanisms such as complement factors and antimicrobial enzymes [103]. Furthermore, infected neutrophils produce chemokines such as MIP1β, which are involved in macrophage attraction [104], the preferential host cells. Shortly after being infected, neutrophils suffer apoptotic death, which in some cases can be regulated by the parasite. The conclusion when different mouse models are studied can vary. In different reports it was observed that the parasite could induce or postpone the death of the neutrophil [105, 106]. However, in both cases, it is clear that infected neutrophils that undergoes apoptosis act as vessels to deliver parasites to macrophages, optimizing macrophage infection [106]. This is due to the high competence of macrophages to recognize and engulf apoptotic cells and to the decrease in macrophage inflammatory activity [71]. In this scenario, PS exposure by apoptotic neutrophils plays an important role in leishmanial infection, since PS is the main ligand to promote both engulfment and regulation of inflammation. Actually, human neutrophils infected with *L. major* parasites are engulfed by macrophages when they are annexin V-positive and therefore are exposing PS at their surface [106]. In this case, PS-exposing neutrophils act as Trojan horses, maintaining viable parasite and transferring them to macrophages [105].

## Conclusions

Albeit apoptotic markers followed or not by apoptotic death, have been observed in unicellular organisms of several different groups of eukaryotes, the present review focuses exclusively on the role of PS exposure and recognition on the natural history of infection by unicellular parasites. The consequences of PS recognition in such events, independently of the origin of the cell exposing the ligand, includes induction of phagocytosis by host cells, infected cell clearance and adherence, induction of anti-PS antibodies as well as inhibition of the host inflammatory response. In the present review, we consider classical apoptotic mimicry when PS is exposed by the parasite itself and non-classical apoptotic mimicry when host cells expose the ligand in the context of the infective process. Table 1 displays both situations. It is important to consider that the cell and molecular mechanisms involved in non-classical apoptotic mimicry are in general better understood than those that are responsible for the classical form of apoptotic mimicry. This is certainly true for the biochemical machinery involved in PS exposure by multicellular host organisms when compared to the mechanism involved in PS exposure by unicellular organisms, in spite of some evidences already obtained for *Leishmania* spp. [39]. This is also the case regarding the molecular structure and the biosynthesis of phospholipids, including PS. As a matter of fact some controversy still remains regarding the presence and distribution of PS among the several differentiation forms of *Leishmania* spp. [107, 108].

**Table 1:** Protozoan parasites in which apoptotic mimicry was described as important for disease establishment and development

	Protozoan parasites				
	*Leishmania* spp	*Plasmodium* spp	*Toxoplasma gondii*	*Trypanosoma cruzi*	*Entamoeba histolytica*
Classical apoptotic mimicry	PS exposure on amastigotes and promastigotes [5, 37, 44, 45]	–	PS exposure on tachyzoites [51, 53]	PS exposure on trypomastigotes [49]	–
Non-classical apoptotic mimicry	PS-exposing neutrophils acting as Trojan horses [105, 106]	PS exposure on infected RBC leading to disease development [75, 76, 84]	–	Apoptotic T cells modulating host macrophages [99]	Phagocytosis of PS-exposing apoptotic host cells [89–91, 93–97]

It is clear that PS recognition is an important feature of host/pathogen interaction, not restricted to pathogenic protozoa, but also involved in the natural history of several important viral diseases.

## Data Availability

Not applicable.

## Abbreviations

iNOS: Inducible nitric oxide synthase
iRBC: Infected red blood cell
KO: Knock out
MCP: Macrophage chemotactic protein
MIP1β: Macrophage inflammatory protein
NADPH: Nicotinamide adenine dinucleotide phosphate
NO: Nitric oxide
PfEMP1: *P. falciparum* erythrocyte membrane protein 1
PS: Phosphatidylserine
ROS: Reactive oxygen species
TGF- β1: Transforming growth fator β1ERK extracelular signal-regulated kinase
